# Effects of high Ca and Mg stress on plants water use efficiency in a Karst ecosystem

**DOI:** 10.7717/peerj.13925

**Published:** 2022-08-17

**Authors:** Rui Qu, Guilin Han

**Affiliations:** Institute of Earth Sciences, China University of Geosciences (Beijing), Beijing, China

**Keywords:** Plant nutrients, Stoichiometry, WUE, Life form, Southwest China

## Abstract

**Background:**

Karst ecosystems are widely distributed in the world, with one of the largest continuous Karst landforms in Southwest China. Karst regions are characterized by water shortage, high soil calcium (Ca) and magnesium (Mg) content, and soil nutrient leaching, resulting in drought stress and growth limitation of plants.

**Methods:**

This study compared nitrogen (N), phosphorus (P), potassium (K), Ca, and Mg of herbaceous and woody plants in a small Karst ecosystem in Southwest China. The indexes of water use efficiency (WUE) were calculated to identify the drought stress of plants in this Karst ecosystem. Meanwhile, the relationship between Ca and Mg accumulation and WUE was evaluated in herbaceous and woody plants.

**Results:**

Herbaceous plants showed a higher content of leaf N (13.4 to 40.1 g·kg^−1^), leaf P (2.2 to 4.8 g·kg^−1^) and leaf K (14.6 to 35.5 g·kg^−1^) than woody plants (N: 10.4 g to 22.4 g·kg^−1^; P: 0.4 to 2.3 g·kg^−1^; K: 5.7 to 15.5 g·kg^−1^). Herbaceous plants showed a significantly positive correlation between WUE and K:Ca ratio (R = 0.79), while WUE has a strongly positive correlation with K:Mg ratio in woody plants (R = 0.63).

**Conclusion:**

Herbaceous plants suffered from nitrogen (N) limitation, and woody plants were constrained by P or N+P content. Herbaceous plants had higher leaf N, P, and K than woody plants, while Ca and Mg showed no significant differences, probably resulting from the Karst environment of high Ca and Mg contents. Under high Karst Ca and Mg stress, herbaceous and woody plants responded differently to Ca and Mg stress, respectively. WUE of herbaceous plants is more sensitive to Ca stress, while that of woody plants is more sensitive to Mg stress. These findings establish a link between plant nutrients and hydraulic processes in a unique Karst ecosystem, further facilitating studies of the nutrient-water cycling system in the ecosystem.

## Introduction

Karst landforms are widespread globally, accounting for 15% of the global land area (Liu et al., 2020b; Zhang et al., 2021). Southwest China possesses one of the largest continuous Karst regions (~550,000 km^2^) in the world (Han et al., 2021; Wang et al., 2019). Karst ecosystems are characterized by low soil formation, shallow soil layer, high permeability, and water shortage (Green et al., 2019; Liu et al., 2021); thus, plants in Karst regions always suffer from drought stress (Geekiyanage et al., 2019). Water use efficiency (WUE), defined by the quantity of water utilized per carbon gain, reflects the plant water strategy under various water circumstances, which is vital for their survival and productivity (Nie et al., 2014; Yan et al., 2016). Leaf δ^13^C data have been proved to best quantify WUE by reflecting both water conditions and the physiological status of plants (Cabrera-Bosquet et al., 2007), since leaf ^13^C discrimination displays a linear correlation with the ratio of intercellular to ambient CO_2_ concentration (Farquhar, Ehleringer & Hubick, 1989). Therefore, leaf δ^13^C data can be a proxy for the WUE of plants.

WUE is influenced by stomatal conductance, photosynthesis, and respiration processes, which are closely related to leaf nutrient concentration (Cabrera-Bosquet et al., 2007; Si et al., 2015; Zhang et al., 2012). Currently, very little is known about the association between WUE and leaf nutrients in the Karst region, though there already exists a few studies on this topic (Yan et al., 2016; Zhou et al., 2016). For example, leaf potassium (K) content affects stomatal conductance, and meanwhile, WUE shows a strong correlation with K content (Cao et al., 2009; Zhao et al., 2007). K is the second most abundant nutrient in leaves after nitrogen (N), essential for plant-water relationships, such as cell osmosis adjustment and stomatal behavior (Seleiman, 2019; Si et al., 2015; Vodnik et al., 2019). However, leaf K shows significantly antagonistic effects with calcium (Ca) and magnesium (Mg) (Haghshenas, Arshad & Nazarideljou, 2018; Zhou et al., 2013). Furthermore, carbonate rocks dominate Karst regions, resulting in Ca and Mg enrichment in soil (Green et al., 2019; Li et al., 2019); thus, the influence of high Ca and Mg stress on the WUE of plants is still unclear. Meanwhile, a lack of essential soil nutrients, such as N and phosphorus (P), limits plant growth and increases the drought stress rate in the Karst region (Carrière et al., 2020; Liu et al., 2021). N and P are two major components of proteins related to energy, participating in the structural compositions of plants (Ågren, 2004; Seleiman et al., 2021b; Yuan & Chen, 2015). Furthermore, the leaf N:P ratio can reflect the nutrient limitation of plants (Jiang et al., 2021; Qiu, Wei & Li, 2013). Additionally, plants play an essential role in nutrient cycling and hydraulic processes, and leaf nutrients (N, P, K) are important indicators by which plants reflect their nutrient status and acquisition mechanisms (Han et al., 2011; Mao et al., 2018).

Therefore, this study analyzed herbaceous and woody plants in a typical Karst region of Southwest China. The goals of this study were to (1) understand the nutrient stoichiometry and limitation of herbaceous and woody plants; (2) examine interactions between WUE and plant nutrition, and (3) discuss the effects of high Ca and Mg stress on the WUE of herbaceous and woody plants in the Karst ecosystem, and further provide information for nutrient cycling and hydraulic processes of plants in a unique Karst ecosystem.

## Materials and Methods

### Study area and sample collection

**Figure 1:**
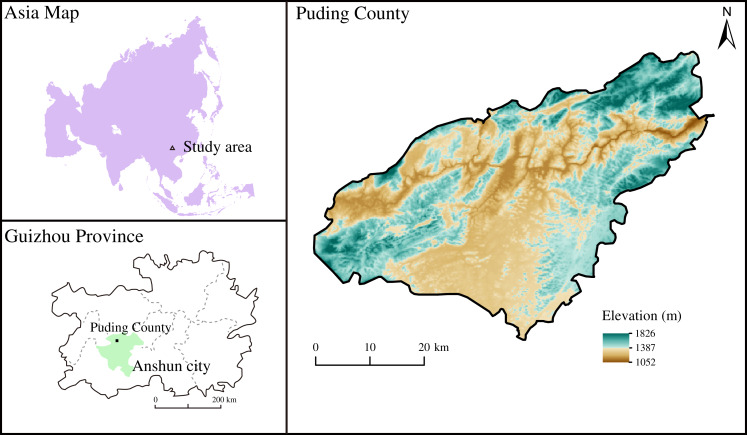
The location of sampling site.

The study area is located in Puding County, Anshun City, Guizhou Province in Southwest China (26°15′ to 26°16′ N, 105°46′ to 105°47′ E) (Fig. 1), a typical Karst area with the dominant dolomite bedrock. Dolomite and limestone from Middle Triassic generated calcareous soil (Han et al., 2020; Zhao et al., 2010) and were further transported and deposited as quaternary soil with high Ca and Mg contents. Subtropical monsoons dominate the climate of the study area, with an annual mean temperature of 15.1 °C and precipitation of 1,400 mm, respectively.

A total of 19 plant samples, including seven types of herbaceous plants and 12 types of woody plants, were collected in this Karst region in June 2016. The herbaceous plants include peanut (*Arachis hypogaea Linn*.), sunflowers (*Helianthus annuus L*.), bracken (*Pteridium aquilinum*), roof iris (*Iris tectorum Maxim*.), taro (*Colocasia esculenta*), Indian chrysanthemum (*Chrysanthemum indicum*), and horseweed (*Conyza canadensis*). In contrast, the woody plants include Chinese firethorn (*Pyracantha fortuneana*), bird cherry (*Prunus padus L*.), oak tree (*Quercus fabri*), Chinese catalpa (*Catalpa ovata*), wheel wingnut (*Cyclocarya paliurus*), prickly wild rose (*Rosa acicularis*), Japanese camellia (*Camellia japonica*), dahurian buckthorn (*Rhamnus davurica*), litsea cubeba (*Litsea pungens Hemsl*.), lobular buckthorn (*Rhamnus parvifolia Bunge*), Chinese ilex (*Ilex chinensis Sims*), and Eucommia bark (*Eucommia ulmoides Oliver*). The target plants are dominant and relatively abundant in the study area, mainly sampled from the upper canopies and with three to five healthy leaves of similar ages at random of the same plants. The collected leaves were rinsed with deionized water (Cascada™, 18.2 MΩ cm). The tissues were cleaned and then freeze-dried. Before elemental analysis, the dried samples were crushed to a powder and passed through a sieve (<150 μm).

### Chemical analysis and calculation of WUE

After grounding into uniformly fine powders, the N contents of leaf samples were determined by an elemental analyzer (Elementar, Langenselbold, Germany) at the Surficial Environment and Hydrological Geochemistry Laboratory of the China University of Geosciences (Beijing). Before analyzing other nutrients, the sample powders were dissolved in 3 mL concentrated HNO_3_ for over 48 h inside a high-pressure steel bomb at 190 °C in an oven. The digested samples were then analyzed for P, K, Ca, and Mg contents by Optima 5300DV ICP-OES (PerkinElmer, USA). The N content was measured by a multi-element analyzer (Vario TOC Cube, Elementar, Langenselbold, Germany) (Zeng et al., 2022). The samples were randomly measured again to ensure the recovery percentage from 95% to 105% (Gao et al., 2021; Wang et al., 2021). All plant species and nutrients data were shown in Table S1 in the Supplemental File. The stable carbon isotopic ratio (^13^C/^12^C) was measured using an isotope mass spectrometer (MAT-253;Thermo-Finnigan, San Jose, California, USA) at the Institute of Geochemistry, Chinese Academy of Sciences. The data of *δ*^*13*^*C* were from Liu, Han & Zhang (2020a). The *δ*^*13*^*C* values were described as the following equation:

(1) $$\delta^{13}C\, (\% _0) = [(R_{sample} - R_{standard})/R_{standard}\times 1,\!000]$$

where *R*_sample_ and *R*_standard_ are the ^13^C/^12^C ratios of the sample and standard of Vienna Pee Dee Belemnite, respectively. The external precision was better than ±0.1‰ after multiple measurements of the standard material. The following formula links Δ^13^C to the ratio of CO_2_ in the leaf intercellular space to CO_2_ in the atmosphere (*C*_*i*_*/C*_*a*_):

(2) $$\Delta^{13}C \,(\% _0) = a + (b - a)(C_{i}/C_{a})$$

where *a* (4.4‰) represents the discrimination against ^13^CO_2_ during CO_2_ diffusion *via* stomata, and *b* (27‰) reflects carboxylation discrimination (Farquhar & Richards, 1984; O’Leary, 1981). The atmospheric CO_2_ concentration *C*_*a*_ was calculated as the following equation (Feng, 1998):

(3) $$C_{a} = 277.78 + 1.350exp(0.01572\times(t-1740))$$

where *t* represents the sampling year, which is 2016 in this study. The WUE can be calculated by the relationship between *Δ*^*13*^*C* and *C*_*a*_ using the following equation (Hietz, Wanek & Dünisch, 2005; Osmond, Björkman & Anderson, 1980; Peñuelas, Canadell & Ogaya, 2011):

(4) $$WUE = A/g_{H_{2}O} = C_{a}(b-\Delta^{13}C)/1.6(b-a)$$

where *A* is the net photosynthesis, 1.6 is the ratio value of leaf conductance to water vapor (*g*_*H2O*_) and CO_2_ (*g*_*CO2*_) (Hietz, Wanek & Dünisch, 2005; Peñuelas, Canadell & Ogaya, 2011).

### Data analysis

One-way ANOVA with the least significant difference (LSD) test was performed to test the significant differences between herbaceous plants and woody plants of N, P, K, Ca, and Mg contents. Pearson correlation coefficient was used to identify the correlations between WUE and K, Ca, and Mg content. The coefficient of *R* square and *p*-value determined the equations of best-fit lines. Statistical analyses were conducted by the IBM SPSS Statistics for Windows, Version 27.0 (IBM, Armonk, NY, USA).

## Results

**Figure 2:**
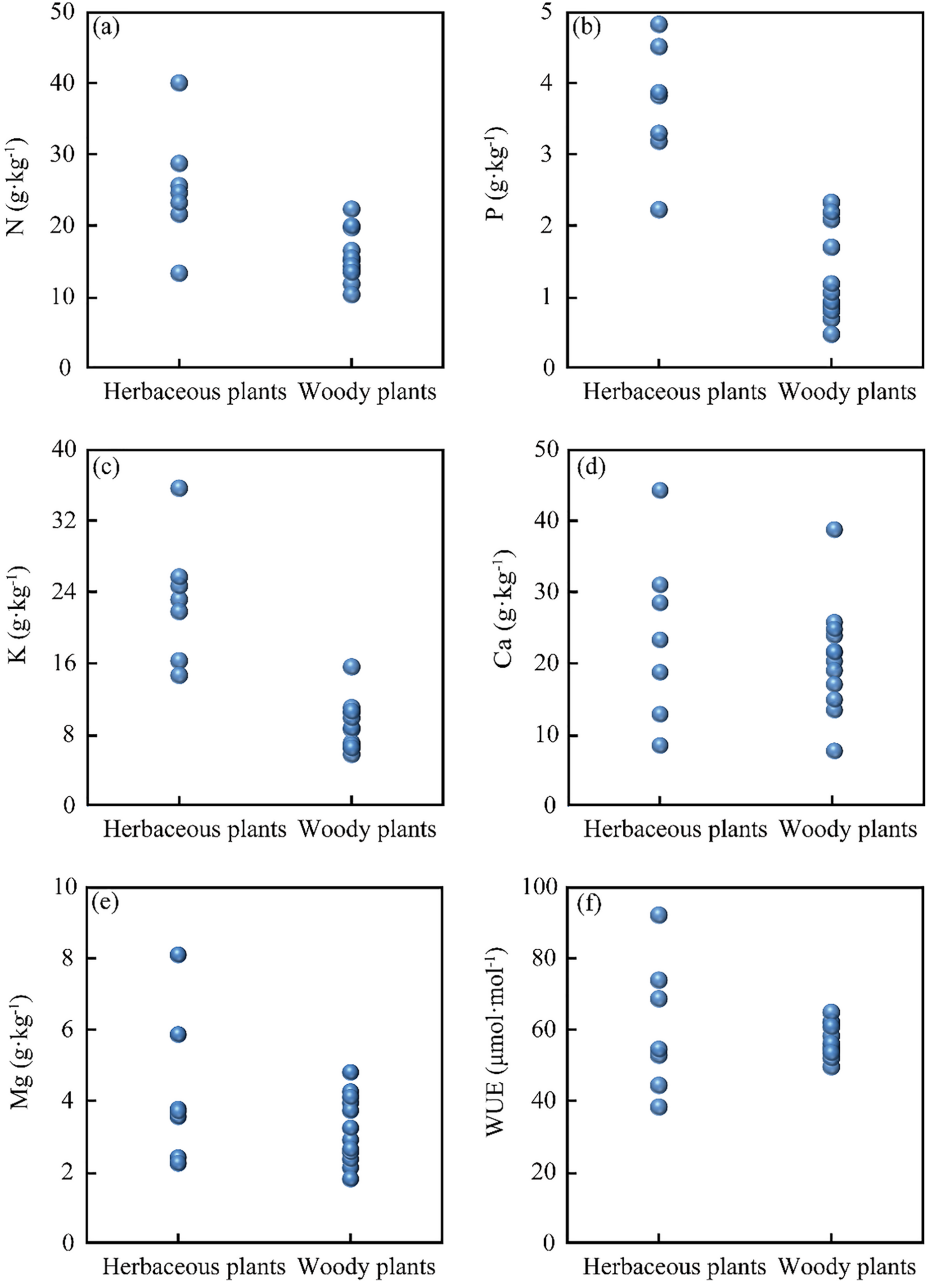
Foliar nutrient content and water use efficiency of herbaceous plants and woody plants: (A) N content, (B) P content, (C) K content, (D) Ca content, (E) Mg content, and (F) WUE.

The leaf nutrients (N, P, K, Ca, Mg) and the WUE of herbaceous and woody plants are presented in Fig. 2. The leaf nutrient contents in herbaceous plants are as follows: N contents from 13.4 to 40.1 g·kg^−1^, P contents from 2.2 to 4.8 g·kg^−1^, K contents from 14.6 to 35.5 g·kg^−1^, Ca contents from 11.3 to 44.3 g·kg^−1^, Mg contents from 2.1 to 8.3 g·kg^−1^, and WUE from 38 to 92 μmol·mol^−1^. In woody plants, the leaf contents are: N from 10.4 to 22.4 g·kg^−1^, P from to 2.3 g·kg^−1^, K from 5.7 to 15.5 g·kg^−1^, Ca from 6.1 to 38.8 g·kg^−1^, Mg from 1.4 to 4.6 g·kg^−1^, and WUE from 49 to 64 μmol·mol^−1^. Overall, these results indicate significant differences in leaf nutrients between herbaceous plants and woody plants in this study area.

## Discussion

### Leaf stoichiometry in herbaceous plants and woody plants

Karst soil is characterized by the scarcity of N and P elements but high Ca and Mg contents (Li et al., 2019). Previous studies reported the soil nutrients of N content (3.1 to 7.3 g·kg^−1^), P content (0.5 to 1.18 g·kg^−1^), K content (27.7 g·kg^−1^), Ca content (3.8 to 34.9 g·kg^−1^), and Mg content (0.9 to 22.6 g·kg^−1^) in Puding County (Kuang et al., 2010; Wang et al., 2018). As shown in Figs.2A–2C, herbaceous plants have higher N, P, and K contents than woody plants. Previous studies have reported higher N, P, K, and Mg contents of herbaceous plants compared to woody plants (Borisade, 2020; Tian et al., 2018), which was attributed to the higher growth and turn-over rates and the rapid decomposition of herbaceous plants (Gilliam, 2007; Güsewell & Koerselman, 2002). Our results corroborated the previous findings that herbaceous plants (short life) contained more N and P contents than woody plants (long life) (Güsewell & Koerselman, 2002; Han et al., 2005). However, there were no significant differences in leaf Ca, Mg content between herbaceous plants and woody plants (Figs. 2D and 2E). This phenomenon was probably attributed to the alkaline soil with the enrichment of high Ca and Mg content in the Karst region (Li et al., 2019; Liu et al., 2021), which limited the growth of plants (Carrière et al., 2020). As shown in Fig. 2F, herbaceous plants exhibited a wider WUE range than woody plants. Woody plants are likely to have deep roots and rely on water from deep soil layers, while herbaceous plants rely on water from shallow soil layers (Nie et al., 2014; Nie et al., 2011; Rose, Graham & Parker, 2003; West et al., 2008). Therefore, under the condition of high soil permeability and water shortage in the Karst region (Liu et al., 2021), woody plants should have more steady water-use strategies under drought conditions (Fig. 2F). In other aspects, the various WUE in plants may be related to K content. A possible explanation is that plants collected osmoticum (K^+^) in response to water stress to raise cell osmotic potential, attracting water into cells (Si et al., 2015). Therefore, herbaceous and woody plants have significantly distinctive nutritional needs, and their WUE reveals quite different features.

**Figure 3:**
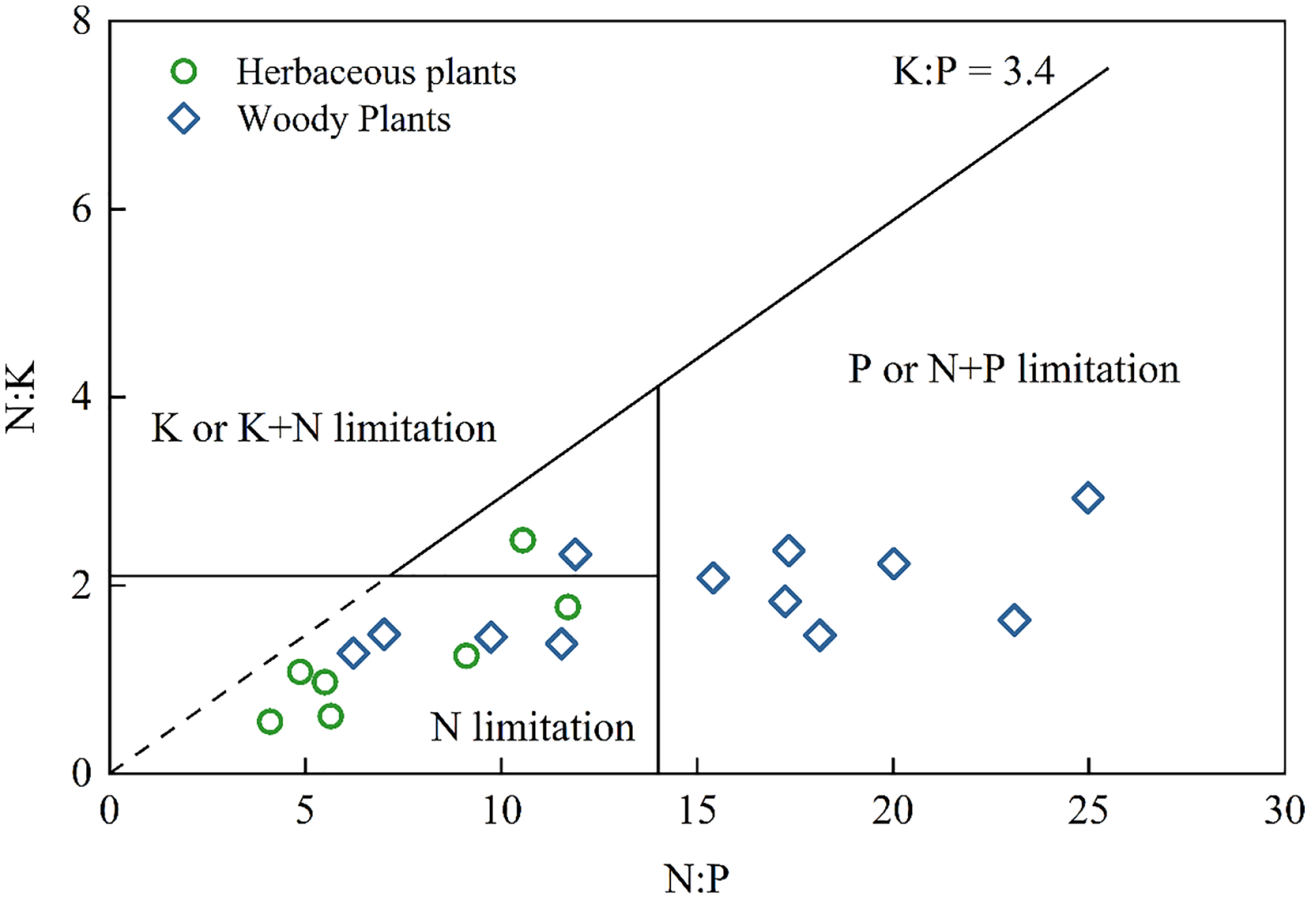
Diagram of nutrient stoichiometry in herbaceous plants and woody plants according to the type of nutrient limitation from Olde Venterink et al., 2003.

Nutrient leaking from thin, calcareous soils in the Karst region may decrease the remaining capacity of soil to deliver essential ecosystem services, further affecting carbon sequestration and food production (Ma et al., 2018; Song et al., 2017). Therefore, it is necessary to understand the type of nutrient limitation in plants. Previous study has suggested several nutrient limitations (Olde Venterink et al., 2003): N limitation (N:P < 14.5 and N:K < 2.1), P or P+N limitation (N:P > 14.5 and K:P > 3.4), and K or K+N limitation (N:K > 2.1 and K:P < 3.4). As shown in Fig. 3, almost all herbaceous plants and several woody plants exhibited N limitation, while the other woody plants were P or N+P limited. These results were consistent with the findings that N limited grassland growth, P limited secondary forest growth, and N+P limited shrub growth in Southwest China (Zhang et al., 2015). None of the herbaceous plants and woody plants suffer from K limitation. Therefore, agricultural activities in this Karst area should apply more N fertilizer to crops. However, the limitation of plant nutrients does not rule out the dilution effects due to the specific Karst environment. For example, K, Ca, and Mg contents can influence the NO_3^−^_ and NH_4_^+^ uptake of plants (Chen et al., 2021) and the antagonistic interaction between K and Ca (Haghshenas, Arshad & Nazarideljou, 2018). Further study should consider using the isotope tracing method to reveal the specific sources of elements and determine the influence of elements on each other.

### Water use efficiency in herbaceous plants and woody plants

**Figure 4:**
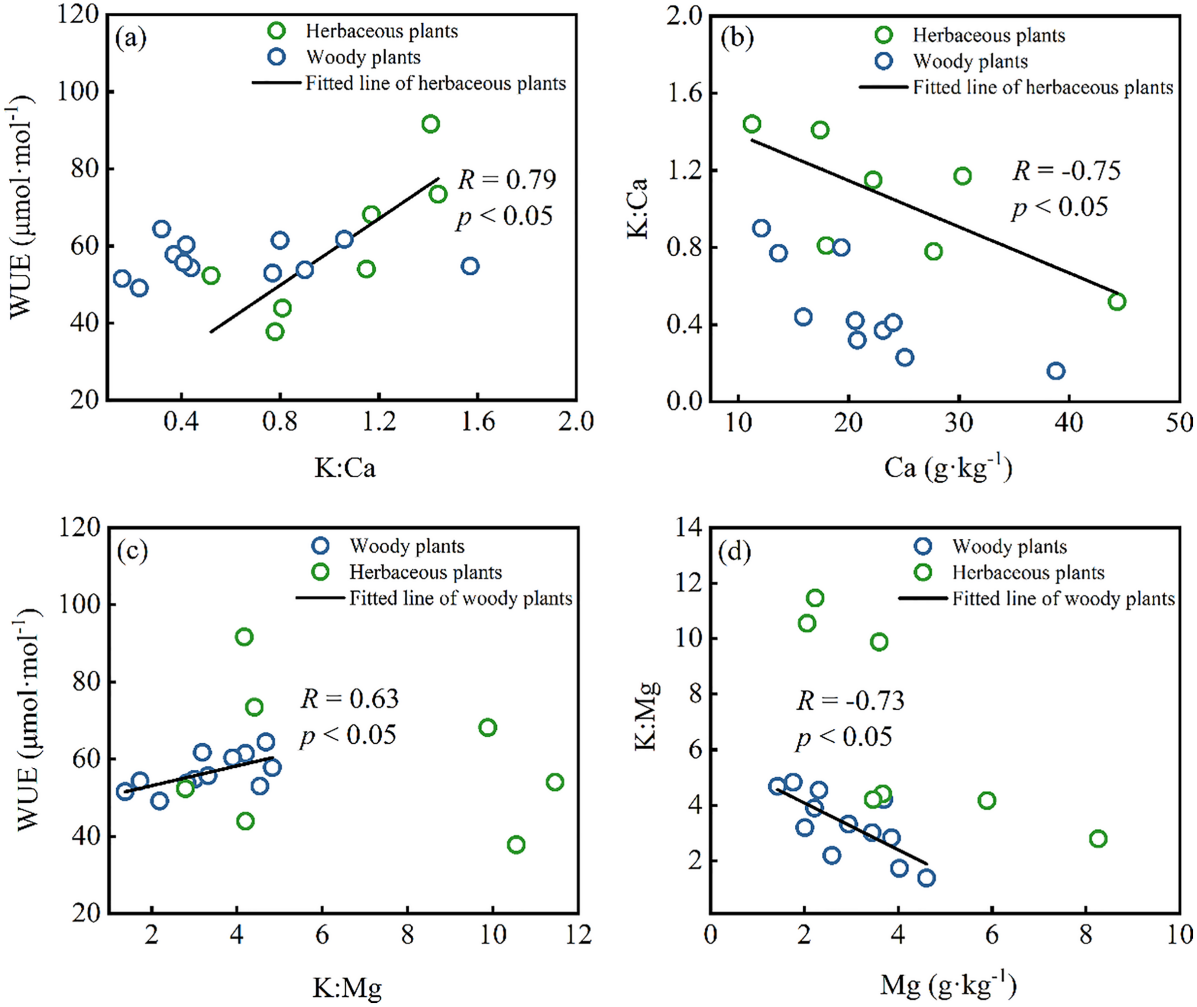
Correlations between leaf nutrients and the water use efficiency (WUE) in herbaceous plants and woody plants: (A and B) K:Ca ratio *vs*. WUE and Ca content; (C and D) K:Mg ratio *vs*. WUE and Mg content.

Drought stress on plants can greatly affect their growth, yield, and grain quality (Seleiman et al., 2021a). Though the precipitation of 1,400 mm is in the study area, water resources in Karst regions are still limited due to shallow soil and high permeability of carbonate rocks, resulting in plants facing drought stress when growing (Liu et al., 2021). The leaf δ^13^C (from −25.3‰ to −30.41‰) of all plants is higher than typical subtropical species with leaf δ^13^C from −31.1‰ to 30.5‰ (Qu et al., 2001), showing plants suffer more drought effect in this area. Under long-term water shortage, plants will employ several strategies in response to drought stress, such as elevating cell osmotic potential or adjusting leaf stomatal conductance (Seleiman et al., 2021a; Si et al., 2015; Vodnik et al., 2019). K plays a critical role in the above functions since K is primarily delivered passively in plant organs through the xylem flow. Regarding leaf cells, guard cells acquire K^+^ and have a higher concentration than nearby cells, and K^+^ outflow causes turgor loss in guard cells (Misra, Reichman & Chen, 2019). Turgor loss in the paired guard cells closes the stomatal gap, which controls water and gases (Melkikh & Sutormina, 2022). Previous studies found a positive relationship between leaf K and WUE (Cao et al., 2009; Zhao et al., 2007), which means high leaf K levels can increase stomatal sensitivity to water stress and lower stomatal conductance (representing higher δ^13^C) (Zhou et al., 2013). However, no significant correlation between leaf K and WUE occurred in the Karst region of this study. On the contrary, WUE showed a strong positive correlation with leaf K:Ca ratio in herbaceous plants (Fig. 4A), implying the effects of Ca on the WUE. Meanwhile, there was a strong correlation between leaf K:Ca raito and leaf Ca content (Fig. 4B), indicating that Ca content mainly controlled the ratio of K and Ca. Similarly, K:Mg ratio showed a strong correlation with WUE and Mg content in the woody plants (Figs. 4C and 4D), indicating the impact of leaf Mg on WUE and the ratio of K and Mg. Under high calcium stress, a large amount of Ca^2+^ influx inhibited the outflow of H^+^ by reducing the activity of plasma membranous H^+^-ATP-ase, which further affected plant stomata, photosynthesis, and transpiration (Sukhova, Akinchits & Sukhov, 2017). Mg plays an essential role in photosynthesis as the central atom of the chlorophyll molecule (Pokharel et al., 2018). Furthermore, the antagonistic effects of Ca and Mg on K in plants have been widely reported (Haghshenas, Arshad & Nazarideljou, 2018; Zhou et al., 2013). Therefore, WUE was affected by the joint influence of leaf Ca, Mg, and K content under high Ca and Mg stress in the Karst region. Additionally, the WUE of herbaceous plants was more sensitive to Ca, whereas the WUE of woody plants was more sensitive to Mg.

## Conclusions

This study provided leaf nutrients (N, P, K, Ca, and Mg) stoichiometry and water use efficiency of herbaceous plants and woody plants in a typical Karst region. Herbaceous plants showed the characteristics of N limitation, and woody plants exhibited P or N+P limitation in the study area. N, P, K content showed significant variations between herbaceous plants and woody plants, while no significant differences occurred in Ca and Mg content due to high Ca and Mg stress in the Karst region. Under high Ca and Mg stress, WUE displayed a strong positive correlation with K:Ca ratio in herbaceous plants and with K:Mg ratio in woody plants, indicating that herbaceous plants were more sensitive to Ca and woody plants were more sensitive to Mg. Our findings provided information on nutrient-water interaction by revealing relationships between leaf nutrients and hydraulic processes in terrestrial plants under high Ca and Mg stress in a unique Karst region, further facilitating studies of nutrient and hydrological cycles in the ecosystem. Further research is required to investigate the water-use strategies of widespread species responding to environmental conditions and explore the water cycle from root to leaf linked to the nutrients.

## Supplemental Information

10.7717/peerj.13925/supp-1 Supplemental Information 1 Leaf nutrients (N, P, K, Ca, Mg) and water use efficiency (WUE) of herbaceous and woody plants.

## Additional Information and Declarations

### Data Availability

The following information was supplied regarding data availability: The raw data are available in the Supplemental File.
